# Monthly Summary

**Published:** 1881-02

**Authors:** 


### MONTHLY SUMMARY.

*Tooth Structure Illustrated.*—Mr. Geo. Fell, of Buffalo, N. Y., gave a description of a series of plates illustrating the structure of the human teeth. In explanation the speaker said these plates constituted a series of enlarged sectional drawings cut transversely, exhibiting the structure, microscopic and general, of a human molar tooth. The average size of the sectional views is about six inches square, beginning with a view of the grinding surface or top view of the crown of the tooth, and successively introducing the structure and conformation of the enamel, cementum, dentine, and pulp cavity, up to the fangs of the tooth as located in the alveolar portion of the superior maxillary bone. The exhibitor gave a very interesting description of the structure of the teeth, aided by the plates, which were marvels of ingenuity and neatness, being constructed of sheets of paper overlapping one another, colored to nature, and so constructed that the various sections could be unfolded and each successive layer of the interior structure of the tooth consecutively exhibited.

Mr. Fell stated that the drawings were prepared from a series of sections of human teeth made by himself sufficiently well to make out the structure with the mic**ɹ**oscrope. His object in preparing them was to afford the medical and dental student another aid to the study of the structure of the human tooth. For the purpose of locating the sections he had prepared a modified copy (side view) of the tooth of Dr. F. G. Lomercier, of Paris, upon which the sections were represented by a series of transverse lines.—*Proceedings of the American Society of Microscopists.*

*Wax Tree*—After numerous essays made in the greenhouses of the Jardin des Plantee, Paris, they have been successful in raising numerous plants of a useful tree, hitherto almost unknown in Europe; this is the Gouing-amadon, commonly known as the wax tree of Cayenne. We have no indications of its botanical definition, whether it is one of the wax berries *Myricas*, or a wax palm. It is said to yield a wax similar to that of the bee, and equally applicable. The culture of the tree is not costly, and on arriving at maturity it is said to yield 50 to 60 lbs. of wax. It is to be tried in Algeria.—*Druggists Circular.*

*India Rubber Deterioration.*—India rubber is subject to two kinds of deterioration and decay. In one instance it tends to become soft, and loses its elasticity, while in the other it becomes friable, yellowish, and resinous in its nature. The last mentioned kind of deterioration has been clearly and indubitably traced to an oxidation of the caoutchouc. This oxidation is tolerably rapid when the caoutchouc exists in a finely divided state, and when it is exposed to damp at the same time; but the alternate damping and drying of the caoutchouc tends more toward its rapid oxidation than does a continual state of dampness. The resinous matter resulting from the oxidation of caoutchouc has been carefully studied by Spiller, who found that a sample of felt, originally composed of cotton fibers and India rubber, had become so far changed during six years as to contain no trace of caoutchouc; but in its place he found a resinous substance resembling shella. The conditions under which the softening of the India rubber takes place are not so well understood, but there is some reason to believe that this is due to incipient oxidation. Ozone oxidizes caoutchouc with extreme rapidity, and the action of ozone on vulcanized India rubber is similar to its action on the unvulcanized material.—Bolas, in *Popular Science Monthly.*

---

*Poisoning by Tincture of Arnica.*—Another fatal case of poisoning by tincture of arnica is reported in the *Lancet.* A man, apparently in perfect health, drank a quantity of tincture of arnica estimated at from two ounces to two ounces and a half. He immediately complained of a sensation of burning in the stomach. Bicarbonate of soda was given, and afterwards some aromatic tincture. By these his sufferings were relieved for some hours, but the pain subsequently returned, and he died thirty-six hours after having taken the poison. This case suggested some experiments as to the irritating character of arnica. An ounce of tincture of arnica was evaporated, and yielded an extract, a little of which, spread over the skin, caused a popular eruption. A larger quantity produced vesication, just as does cantharides. The residue found in the stomach of the poisoned man, evaporated and then applied to the skin, produced effects exactly the same as those caused by the evaporated tincture.—*Druggists Gazette.*

